# *Neospora caninum* Infection in Cattle in the Province of Kohgiluyeh and Boyer Ahmad, Southwest of Iran: Seroprevalence and Molecular Assessment

**DOI:** 10.1155/2021/4258513

**Published:** 2021-12-24

**Authors:** Atefeh Darijani, Nasir Arefkhah, Sepehr Shahriarirad, Sina Zoghi, Mehdi Namavari, Abdolali Moshfe, Marzieh Zaraei, Bahador Sarkari

**Affiliations:** ^1^Department of Parasitology and Mycology, School of Medicine, Shiraz University of Medical Sciences, Shiraz, Iran; ^2^Cellular and Molecular Research Center, Yasuj University of Medical Sciences, Yasuj, Iran; ^3^Student Research Committee, Shiraz University of Medical Sciences, Shiraz, Iran; ^4^Shiraz Branch, Razi Vaccine and Serum Research Institute, Agricultural Research, Education and Extension Organization (AREEO), Shiraz, Iran; ^5^Basic Sciences in Infectious Diseases Research Center, Shiraz University of Medical Sciences, Shiraz, Iran

## Abstract

**Introduction:**

Among the protozoa of veterinary importance, *Neospora caninum* is responsible for large economic and productive losses in cattle herds. Dogs are being considered the definitive hosts of the parasite. *Neospora caninum* has gained considerable attention through its role in the etiology of bovine abortion. The current study aimed at assessing the status of *Neospora* infection in cattle in Boyer-Ahmad County in Kohgiluyeh and Boyer-Ahmad province, southwest of Iran.

**Methods:**

In this cross-sectional study, 150 cattle blood samples were collected and samples were screened for *N. caninum* antibodies using a modified direct agglutination test (MAT). For the same samples, 130 buffy coats were collected and tested, by PCR, for the presence of *N. caninum* DNA, targeting the Nc-5 gene.

**Results:**

Anti-*N. caninum* antibodies were detected in the sera of 49 out of 150 cattle which is corresponding to a seroprevalence rate of 32.7%. *Neospora* DNA was detected in buffy coats of 26 out of 127 (20.47%) cattle. Even though *Neospora* infection was more common in females and in five-year-old cattle by serology and molecular methods, yet there was no statistically significant difference between age, sex, and *Neospora* infection in both molecular and serological methods (*p* > 0.05).

**Conclusion:**

Findings of the current study indicate a high rate of *N. caninum* infection in cattle of Boyer-Ahmad region in the southwest of Iran. This issue should be further investigated and the prevention and control of this parasite in livestock, due to the high financial burden of this parasite in the livestock industry, should be considered.

## 1. Introduction

Neosporosis is an important disease in dogs and cattle with a global prevalence [1]. This disease is known as one of the main causes of abortion in cattle and neuromuscular paralysis in dogs [2]. The causative agent of neosporosis is an obligate intracellular protozoan and a member of the phylum Apicomplexa. One of the most important genus and species of which is *Neospora caninum* [3]. In cattle, the two main routes of *Neospora* infection are vertical transmission from mother to fetus, as well as transmission through ingestion of contaminated water or forage with oocysts excreted in dog feces [1]. Studies in different areas of the world show that a high percentage of aborted fetuses in cattle is due to infection with this parasite [4–6].

*N. caninum* causes miscarriage in cattle, and most miscarriages occur in the second trimester of pregnancy. The embryos are often autolyzed and may become dead or mummified in the uterus or even reabsorbed. Abortions occur throughout the year, both in the first and in subsequent deliveries [2]. Sometimes fetuses are born prematurely or seemingly healthy, without clinical signs [2].

Neosporosis is a common infection in livestock in Iran. According to a recent meta-analysis, the overall seroprevalence of bovine neosporosis in Iran ranges between 3.8 and 76.2% [7]. In a study by Sadrebazzaz et al. in Mashhad, East of Iran, 15.19% of cattle were found to be infected with *N. caninum*, and the rate of abortion in positive cattle was significant [8]. In another study in the same area, Razmi et al. reported that 46% of evaluated cattle are seropositive for neosporosis and that 85% of seropositive cattle had a history of abortions [9]. In a study in Kerman, southeast of Iran, on 285 bovine sera, *Neospora* infection was detected in 36% of the studied cattle [10]. In Ranjbar et al.'s study on aborted dairy cattle in Garmsar city, southeast of Tehran, 38.5% of studied animals were positive for *N. caninum* and there was a significant association between the prevalence of *Neospora* infection and the frequency of abortion in the studied cattle [11]. Rafati and Jaafarian's study on 100 aborted bovine embryos in Shahrekord in the southwestern part of Iran revealed that 11% of the samples are infected with *Neospora* parasite, by molecular method [12]. Ansari-Lari et al. reported a seroprevalence of 30% for *Neospora* infection in cattle in Shiraz, south of Iran [13].

A serological study of bovine neosporosis by Sengupta et al. in India revealed that 10% of the samples were infected with *N. caninum* and that there was a significant association between abortion and serum infection with *Neospora* [14]. A study in Argentina by Fort et al. on 4334 bovine serum showed that 302 (6.9%) of the cases were serologically positive for *N. caninum* infection [15].

Due to the high economic importance of this disease and also the existence of very different climates in different parts of Iran and the lack of sufficient information about this disease in livestock in different parts of the country, the present study is aimed at evaluating the status of *Neospora* infection in cattle in Boyer-Ahmad County in Kohgiluyeh and Boyer-Ahmad province, southwest of Iran, by molecular and serological methods.

## 2. Materials and Methods

### 2.1. Sample Collecting

**Figure 1:**
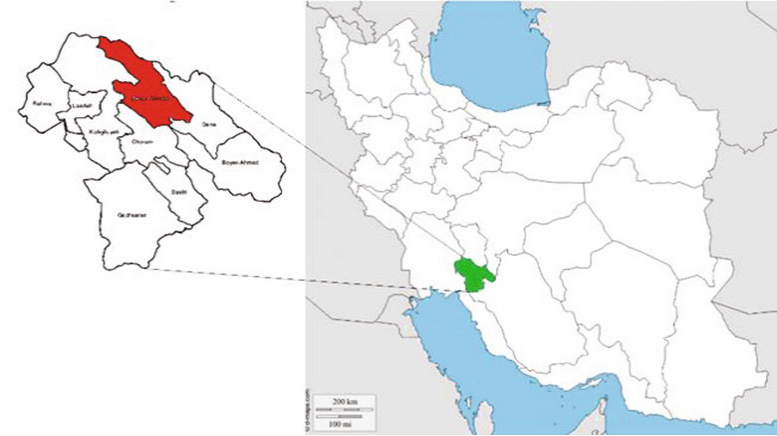
Map of the study area showing Kohgiluyeh and Boyer-Ahmad province and Boyer-Ahmad County in southwest of Iran.

In this cross-sectional study, 150 blood samples were obtained from cattle of Boyer-Ahmad County in Kohgiluyeh and Boyer-Ahmad province (Figure 1). The province is located in the southwest of Iran, where due to favorable weather conditions the prevalence of some of parasitic infection is substantial. Previous studies have reported a significant prevalence of helminth and protozoan infections in human as well as among livestock in the area [16–20].

The studied cattle in the current study were traditionally kept in stables and grazed from open fields and meadows. Information including age, sex, pregnancy status, and history of abortion was recorded in a predesigned datasheet. Of 150 cattle, 82 (54.7%) were male and 68 (45.3%) were female with no history of abortion. The studied cattle were between 1 and 8 years old, and the most common age group was the 5-year-old group, which included 23% of cases. The serum and buffy coat of the samples were separated and stored at -20°C until use. To do that, bloods were centrifuged (1000 *g* for 15 minutes). After centrifuge, at the junction of the plasma and red cells, there is a thin whitish or buffy coat layer. Serum, top layer, was first separated, and then, using a pipette, the buffy coat was collected.

### 2.2. Serological Evaluation of the Serum Samples

#### 2.2.1. Preparation of Neospora Antigen

Vero cells were cultured in RPMI culture medium, containing 10% fetal calf serum, 100 IU/mL of penicillin, 100 *μ*g/mL of streptomycin, 50 *μ*g/mL of gentamicin, and 25 *μ*g/mL of amphotericin. The *N. caninum* Nc1 isolate, provided by Razi Vaccine and Serum Research Institute of Shiraz Branch, Shiraz, Iran, was cultured in a Vero cell line. The culture was examined daily, and when 80 to 90% of the cell destruction by the parasite was observed, the Vero cells along with the tachyzoites were removed from the culture flask. The suspension was immediately washed 3 times, with PBS, and the supernatant was discarded. The tachyzoite pellet was washed in 2-3 mL of 37% formalin, and the concentration of formaldehyde was reduced to 6% with PBS washing solution and the obtained tachyzoite kept at 4°C, overnight for fixation. Then, formalin was removed by washing the sample with PBS. Alkaline buffer (BABS, pH: 8.7) was added to the tachyzoite suspension and that the final concentration of the tachyzoite was adjusted to 30,000 to 40,000 cells/*μ*L.

#### 2.2.2. Modified Agglutination Test (MAT) for Detection of Anti-Neospora IgG Antibodies

MAT was performed as previously described by Tavanaee and Namavari [21]. Briefly, 95 *μ*L saline was added to the first well of a 96 U-shaped microplate, and 50 *μ*L was added to the rest of the wells. Then, 5 *μ*L of cattle serum was added only to the first well of the plate, and from that well, 50 *μ*L was transferred to the second well and continued in the same way until well 12 and the last 50 microliters were discarded. Then, 25 *μ*L of 2ME solution was added to all wells and mixed thoroughly by shaking the plate for 4-5 minutes. Antigen (50 *μ*L) diluted in borate buffer was added to each well, and the plate was shaken for 4-5 minutes as before. The plates were incubated in a wet chamber for 24 hours at room temperature. Finally, the plate was examined for the formation of agglutination under a loop microscope. Rabbit hyperimmune sera, raised against *Neospora* antigens, were used as positive control, and normal rabbit serum was used as the negative control.

### 2.3. Conventional PCR for Amplifying the 340 bp Fragment of the Neospora Nc-5 Gene

The genomic DNA from 127 cattle buffy coat samples were extracted, using a commercial Tissue Genomic DNA Extraction Kit (Favorgen Biotech Corp., Taiwan; FATGK001), following the manufacturer's guidelines. A conventional PCR was performed to amplify a 340 bp fragment of the Nc-5 gene of *N. caninum*, using the NP6 forward (5′-CAGTCAACCTACGTCTTCT-3′) and NP21 reverse (5′-GTGCGTCCAATCCTGTAAC-3′) primers.

A PCR reaction volume of 25 *μ*L that consisted of 12.5 *μ*L of 2x Taq PCR mix (Amplicon, Odense, Denmark), 0.5 *μ*L of each primer (10 pmol/*μ*L), 30 ng of template DNA, and 11.5 *μ*L of ddH2O was prepared. The PCR temperature profile was one cycle at 95°C, 5 min, 30 cycles at 94°C, for 50 sec, 55°C for 30 sec, 72°C for 50 sec, and one cycle at 72°C, 4 min. In each run of the experiment, positive (*N. caninum* DNA) and negative (double-distilled water instead of template DNA) controls were included [22].

For separation of the DNA product, a 1.5% agarose gel in TAE solution was prepared and the PCR product was stained with GelRed nucleic acid gel stain for visualization under a gel documentation device.

### 2.4. Statistical Analysis

In this study, SPSS (ver. 20) software (SPSS, Chicago, IL, USA) was used to analyze the findings of the study. Chi-square test (*χ*^2^) was used to determine the association between the studied qualitative variables.

## 3. Results

Anti-*Neospora* antibodies were detected in sera of 49 out of 150 cattle corresponding to a seroprevalence rate of 32.7%. Even though the infection was more prevalent in five-year-old cattle, nevertheless, the differences between age and *Neospora* seropositivity were not statistically significant (*p* > 0.05).  Table 1 shows the features of the cattle and relative seropositivity to *Neospora* in Kohgiluyeh and Boyer-Ahmad province, southwest of Iran.

**Figure 2:**
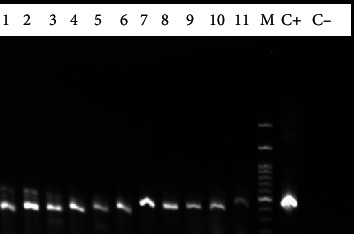
PCR product electrophoresis of the 340 bp fragment of the Nc-5 gene of *N. caninum* on 1.5% agarose gel. Lane 1-11 samples from cattle buffy coat; lane M: 1 kb molecular weight marker; lane C+: positive control; lane C-: negative control.

Infection was more common in male cattle, yet the differences between sex and seropositivity to *Neospora* were not statistically significant (*p* > 0.05). PCR detected the *Neospora* DNA in the buffy coat of 26 out of 127 (20.47%) cattle (Figure 2).

The five-year-old cattle had the most (29%) positive molecular cases of *Neospora* infection, yet there was no statistically significant association between molecular positivity and age of the studied cattle (*p* > 0.05). Also, molecular positivity with *Neospora* was not associated with the sex of the evaluated cattle. Considering the agreement between the serological and molecular methods, 20 seropositive cases were PCR positive. The kappa coefficient test showed a moderate level of agreement (*k* = 0.73) between seroprevalence and molecular infection to *Neospora* in the animals.

## 4. Discussion

Neosporosis is a worldwide disease in which dogs and canines are definitive hosts in its life cycle [1]. Bovine neosporosis has been reported in different parts of the world, and it has been shown that 12-42% of cattle with a history of abortion have been infected with *N. caninum* [23].

The status of cattle infection with *Neospora* in many parts of Iran is unknown. Due to the importance of this parasite, it is worthwhile to determine the status of this parasitic infection in different species of animals, including cattle. These data are important for designing the control programs and to reduce the financial burden imposed by this parasite. The present study was conducted, for the first time, in the southern regions of Iran to find out the status of neosporosis in cattle by serological and molecular approaches and this can be considered as the novelty of this study.

A total of 150 bovine blood samples were examined, using the MAT serological assay to detect *N. caninum* antibodies, and 49 cattle (32.7%) were found to be infected. Moreover, *N. caninum* DNA was detected in the buffy coats of 26 out of 127 (20.47%) cattle, 20 of them were seropositive. The kappa coefficient test showed acceptable agreement between seroprevalence and molecular infection to *Neospora* in the studied animals. The overall seroprevalence of bovine neosporosis in Iran is reported to be 23.6% [4]. The findings which show a high prevalence of *Neospora* in the studied animals are in line with this report. In addition, in another study conducted in Fars province, which is located in the neighborhood of Kohgiluyeh and Boyer-Ahmad province, the prevalence of *Neospora* infection in cattle was reported to be 30% [13]. These studies indicate a high rate of infection with this parasite in cattle in Iran. A higher prevalence of *Neospora* (46%) has been reported from Mashhad in the East of Iran by Razmi et al. [9]. Also, higher prevalence for bovine neosporosis has been reported from other countries including Poland (56%) and Brazil (47.36%) [24, 25].

In the present study, *Neospora* infection was detected by the molecular method in a significant number of cattle. Although this level of infection is consistent with the amount obtained by the serological method, the high rate of infection detected by molecular method in cattle cannot be simply justified. In most studies on *Neospora* infection in different hosts, the prevalence of *Neospora* by the molecular method has been much lower than that reported by the serological method. The same is true in our study, but the rate of infection by molecular method in our study is relatively high. In Duarte et al.'s study regarding the serological and molecular detection of *N. caninum* in human umbilical cord blood, the prevalence of *Neospora* infection was 24% by serological method and 1% by molecular one, using the umbilical cord blood [26]. However, this study utilized the umbilical cord blood for DNA extraction, and a low level of infection in a blood sample in neosporosis is not unexpected. Amdouni et al. in Tunisia also reported a 25% seroprevalence and a 12% molecular prevalence for *Neospora* infection in sheep, although the researchers performed the molecular experiment on the semen specimens [27]. A study in Shahrekord, southwestern Iran, revealed *Neospora* DNA in 11% of the aborted bovine embryos by molecular method [12]. A study by Hariri et al. detected *Neospora* antibodies in the sera of 6.7% of dogs in southern Iran, but none of them were positive by molecular method [28]. In the mentioned studies, samples other than buffy coats have been used to identify the parasite DNA, while in the present study buffy coat sample that seems to be a more suitable specimen has been used. This may partly explain the high molecular prevalence of *Neospora* detected in the present study.

In the current study, both molecular and serological methods were applied for the detection of *Neospora* infection in the studied cattle. For molecular evaluation, the Nc5 gene was targeted which is highly specific and excludes other species of toxoplasmidae family [29]. This can be considered as another strength of the present study. In addition, the presence of parasites' DNA in the animal samples indicates the current state of Neospora infection in the animals.

Furthermore, in the current study, serological method was used to assess the seroprevalence of neosporosis in the cattle. So far, several serological methods have been introduced to detect *Neospora*-specific antibodies in animal milk or serum, including ELISA, indirect fluorescent antibody tests (IFAT), and *N. caninum* modified agglutination test (MAT). These tests are commonly used in epidemiological studies, due to the fact that they detect both previous and current infections in animals and their positiveness indicates previous or recent infection with the parasite.

In the present study, the studied cattle grazed freely on pastures in rural areas where they were in constant and close contact with dogs, the definite host of the parasite. On the other hand, keeping dogs in rural areas including the area where the current study was undertaken, particularly in families with livestock, is common. Therefore, it can be postulated that the high level of *Neospora* infection in the studied cattle is linked to their close contact with the dogs.

## 5. Conclusion

The findings of the present study showed a high rate of *N. caninum* infection in cattle in Boyer-Ahmad region in the southwest of Iran. This issue should be further investigated and the prevention and control of this parasite in livestock, due to the high financial burden of this parasite in the livestock industry, should be considered. Further studies on the prevalence of *Neospora* in other livestock in the region, as well as sequencing of PCR-positive samples and determining the genotype of the parasite in the region, are recommended.

## Figures and Tables

**Table 1:** Features of the cattle and relative seropositivity to *Neospora* in Kohgiluyeh and Boyer-Ahmad province, southwest of Iran.

Characteristics	Frequency		Positive for anti-*Neospora* antibodies		Positive by molecular method (PCR)	
	No.	Percent (%)	No.	Percent	No.	Percent
Age^∗^						
1	32	22.2	11	34.37	5	15.6
2	9	6.25	2	22.22	1	11.1
3	13	9.02	6	46.15	2	15.4
4	11	7.6	3	23.8	1	9.1
5	34	23.6	9	26.47	10	29.4
6	30	20.8	10	33.33	6	20
7	14	9.7	5	35.7	2	14.28
8	1	0.69	1	100	0	0
Sex						
Male	82	54.6	27	32.9	14	17.07
Female	68	45.33	22	32.3	12	17.64
Pregnancy						
Pregnant	43	65	13	30.2	7	16.27
Not pregnant	107	35	36	35.5	20	18.69

^∗^Age of a few of the animals was missing.

## Data Availability

The nominal and ordinal data used to support the findings of this study are available from the corresponding author upon request.
